# “See the half-filled glass and move forward” parental experience of a single mother of two daughters with cognitive disabilities

**DOI:** 10.3934/publichealth.2018.1.64

**Published:** 2018-03-27

**Authors:** Rivka Hillel Lavian

**Affiliations:** Head of M.Ed, Special Education Program, Levinsky College of Education and Inbal Ben Haim, M.Ed.

**Keywords:** cognitive disabilities, single mother, parental experience, narrative research

## Abstract

The aim of this research is to give voice to a single mother of two grown up daughters with cognitive disabilities in order to examine her parental experience. The narrative approach is used in this study. The research tool is an in-depth narrative interview. The interview was recorded and transcribed and the findings divided into key themes that were analyzed in a holistic fashion combining formative and content related aspects. The research finds that this special type of parenting is complex and full of challenges. The mother adapted ways of coping that helped her on her parental journey. Her means of coping were cognitively produced and focused on emotions, with three guiding principles throughout: Seeing the glass as half full, relating to her daughters as normal and the desire to look after her daughters herself and not move them to an external framework. It appears that her optimistic personality influenced her positive parenting style. The research also found that support provided by nuclear family contributed to reinforcing the mother's internal resources and enabled her to maintain a balance between caring for her daughters and developing a personal life and even a new relationship. We hope that insights from this study will enable educational and professional staff to provide appropriate and effective support to mothers of children with cognitive disabilities and consequently create a platform for fruitful and effective collaborations between them and educational and welfare institutions as well as framework that accompany their children after their school years.

## 1. Introduction and literary review

Birth of a new baby directly influences family balance, and even more so when the new baby has cognitive disabilities. Families experience crises from the moment that they suspect any disability, and later during consultation and diagnoses phases and when receiving a diagnosis [1]. Organizational structure, balance and dynamics continue to change when families grow and additional children are born [2]. The birth of a child with special needs creates an unusual operating system, shattering expectations, physically overloaded, unequal division of parental resources, feelings of embarrassment and failure, self-blame and more [3].

Parental adjustment processes have been described through different research perceptions. Until the 1970's, the literature was dominated mainly by models representing parents' reactions and emotions regarding their children with special needs as a development process with a number of pre-known stages: From a stage of shock and bereavement, refusal to accept the diagnosis, anger, guilt, fear and depression and finally acceptance [4]. Since the 1980's, studies have been presented in professional literature contradicting the stage theory. It is rather an infinite process of changes and transitions in parents' lives, demanding over and over again internal work, adjustments and coping [5]. The process is characterized by continual fluctuations between acceptance and non-acceptance paving the way to parting from the dream and enabling parents to see their children's disabilities as part of reality. Salomon [6] found that the need for quick reorganization resulting from the addition of a child with special needs to a family, is not immediate. Over time, this need permeates and consequently a great loss is recognized, loss of a child that parents had expected, acknowledging the idea that their child is not like other children giving up on his/her obvious development. The mourning phenomenon returns at points in time, particularly at transition stages in children's and adults' lives such as: Starting kindergarten, special education framework and more. These transitions remind parents how different their child is from others and therefore are perceived as particularly distressing for parents [7].

Family members who care for children with special needs have reported many difficulties and a lesser quality of life in comparison to parents of children without disabilities. In the literature, it is customary to differentiate between subjective and objective burdens amongst family members. Subjective burden refers to individual evaluation of damage, the extent to which the situation is perceived as burdensome and its consequent emotional distress [8]. For example, it has been found that parenting a child with delayed development negatively influences parents' functioning in the early years because it necessitates unending investing in the child, alongside less rewarding interactions [9].

Mothers of children with special health care needs have perceived their lives as more difficult and have reported more symptoms of depression and higher levels of melancholy, distress fear in comparison to mothers of children without special needs [10]. They explained these differences on the background of pressurizing events that mothers have experienced such as repeated sudden hospitalizations and special medical treatments. In addition, reduced financial, time and strength resources are available to the family as a whole. It was found that parents with adaptive abilities that allowed them to adjust quickly to issues of caring for a child with special medical needs, positively, with no connection to the severity of the child's condition [11]. It has also been found that the birth of a child with special needs, whose appearance indicates his/her disability, makes it even more difficult for a family to cope, in contrast to children with special needs whose appearance does not invite second looks from others [3].

An objective burden is defined as the price a family is expected to pay as a result of its sick or disabled child. With regard to employment, many parents are expected to give up on going out to work, they are limited in type of work and the amount of time they can be away at work in order to look after their child [12]. In addition, greater expenditure is needed to care for their child.

Additionally, parents of children with special needs are forced to give up or reduce their leisure and social activities, both because of a lack of money and/or time and the stigma that their child's disability carries. Parents encounter social pressures, especially in situations where their children show unexpected behaviors such as screaming etc. In order to avoid these situations, they avoid social activities and avoid meeting friends [1].

Alongside traditional perceptions that view mothers as having responsibility for bringing up and nurturing their children, a model is recognized today in which women work outside the home and their role is varied and open and closer to that of men. Nevertheless, there is a high expectation of mothers to provide essential and devoted care to their children. This expectation is even greater if their children have special needs and consequently the effect on mothers' spiritual resources [13]. Studies have shown that mothers of children with special needs are in greater danger of experiencing pressure, anxiety and depression and to have lower emotional, psychological strengths than fathers have [14]. In a study that examined the extent of pressure and fear experienced by mothers of teenagers and adults with delayed development (FXS), by testing the levels of Cortisol (stress hormone) found in their bloodstream. This study confirmed that mothers of children with these disabilities, experienced greater psychological stress and pressure than mothers of children with other types of disability, especially when accompanied by behavioral disabilities. In contrast, it was found that mothers of an additional disabled child, the level of Cortisol was lower in the morning [15]. A low level of Cortisol is the biological signature of what is found in research literature, with regard to feelings of tiredness and fatigue amongst people who experience chronic tension [16]. The widespread assumption is that fathers are less affected than mothers, because generally they carry less of the daily burden of bringing up the child and their possibilities for self-fulfillment outside the home are less harmed. However, there are studies that have found situations in which levels of tension and pressure are identical in fathers and mothers, in periods close to diagnosis, in infancy [13]. The explanation for this is the balanced distribution of responsibility between mothers and fathers.

## 2. Coping modes

In the last decade the Modes of Existence theory [17] has developed. According to this theory, existence is a most important component in Scrutcutal Cognitive Modifiability (SCM), and it serves as a particularly worthwhile addition to adjustment mechanisms, instigating and switching to different states created in humans. On the basis of this model's ideas, we will build a theory based on four modes in which parents of children with special needs cope [18]:

Emotion focused mode: Every expression of emotion that parents feel from the moment of expectation until confirmation of their child's disability. In addition, the feelings accompany parents at every crossroad in their child's life. The very fact that emotions are expressed helps to cope.

Action focused mode: The need to do something, including searching for answers in social and professional circles out of a desire to feel in control of their situation.

Thought focused mode: Turning to intellectual processes, expanding the knowledge of their child's disability, while exercising self-criticism with regard to steps they have taken and reaching conclusions that affect the direction and progress of their coping process.

Values focused mode: A mode of coping that relies on people's value perceptions, which are the foundations of their feelings and thoughts and guide their way in the world. Focus on values is the basis of all aforementioned strategies, as a north star that guides their way.

Tiferet and Elitzur [19] identified religious belief as another coping mode that could be a source of comfort and hope and showings a way of coping.

Despite the fact that parents of children with special needs experience emotional distress, pressures of parenthood and even symptoms of depression, most families tend to explain their family function and quality of life as similar to those without children with disabilities [20]. Antonovsky [21], founder of the salutogenic theory, stated that attention should be focused on healthy aspects and the power sources of personal and familial networks. He emphasized people's spontaneous and inherent coping powers and environmental influences. A study that examined mothers of children with delayed development, who conduct themselves effectively despite enormous challenges, found three defense factors that are likely to affect the mother's parenting: Education, health and optimism [22]. A mother's education is likely to provide her with cognitive resources that will help her provide positive parenting. While at the same time good health is linked to doing energies. In addition, people with high levels of optimism, are more able to adjust to life's events. Mothers' optimism enabled them to maintain positive parenting and cope with situations that arise because of their role as mothers of children with disabilities. Characteristics such as: Emotional autonomy, personal ability and responsibility, moral commitment and flexibility were also found [2].

Today we understand that over and above personality factors, many parameters are mutual partners in family functioning. Mishori [18] identified a number of factors such as: A father's place in a family, relationships within a family, significance of the social environment, events that take place in the lives of every person and qualities of parents and every member of a family. Among these factors are parental image, whether parental approach is conflicting or collaborative, their levels of availability, and their sense of self-efficacy. Baker et al. [23] found that satisfaction with marriage and family unity constituted support and encouragement despite the many difficulties associated with bring up a child. One must remember that these are not isolated factors, but there are reciprocal relations between them and a child's character and characteristics [2]. Another factor that affects its ability to cope is a family's ability to rely on external and internal resources. It was found that when parents include and talk to their close family, relatives and friend, their internal resources are reinforced [24]. A further source is using the various external sources that are available for diagnoses, care and help [25]. Barker et al. [15] argued that mothers adjust to their non-normative parenthood with time and the help of accumulated experience.

Grant et al. [26] developed a model that deals with factors that affect the durability of families of children with disabilities. The factors were divided into three categories: Environmental level—political, cultural, social and financial influences. Individual level—search for meaning, desire for control, existence of identity values. Family level—moral codes, values and culture, conditions of internal and external love and support (from close and wider family circles).

The idea of balances in parenthood “can serve as a new prism for observing parental experiences and coping instead of traditional observations that judge and categorize as positive or negative. It seems like that is one of the central challenges of parenthood, a challenge that continues and renews itself all the time as changes takes place in a child, a parent, the relationship between them and life's circumstances” (translated from [27]).

Families are the main care providers for children with special needs. Even when children are entitled to different services and receive them from different bodies, family members are those who are in contact with service networks and are those who worry about having these services provided [28]. Because of the multiplicity of service providers in this area, the burden faced by parents in their attempts to deal with the confusion in services and rules of entitlement is not easy. In order to take full advantage of services given, parents must have a comprehensive knowledge of what is available and operate effectively in order to coordinate between them and guarantee care for their child in the transition between frameworks. Parents of children with special needs report that there is a lack of information with regard to rights and services as well as types of disability or problems with their children. However, in many cases, family members are likely to come up against bureaucratic difficulties [7]. Parents who receive assistance, relief or benefits from state institutions reported their positive contribution to bringing up children with developmental delays [29].

In the last decade, recognition that “cultivating partnerships based on collaboration with parents leads to early resolution of conflicts and prevents expensive steps such as: Mediation, hearings, due process and litigation in court” (translated from [30]). Nevertheless, there is a gap between experts' wishes and practice and collaboration between experts and parents. There is a lack of research understanding about collaborative components between multi-disciplinary professional teams and parents in general and sensitive and highly stressful meetings in particular.

Very little is known about parental decision-making processes to seek out of house frameworks for their children with developmental disorders. Parents have reported difficulties in fulfilling their parental duties towards their children because they are not under their direct supervision [31]. Despite the difficulties in bringing up children with special needs, concern for their health, economic burden, fear of social difficulties, most parents testify to a positive and optimistic perception of and satisfaction with their lives.

Nevertheless, there is a fear of the future. Despite this, most parents have yet to examine the possibilities for their children in the future. One can interpret this finding as a further factor that affects family durability. Parents of children with special needs have to deal with the present, hoping to return to sound functioning and routine, and they are not interested in examining or dealing with future problems [25].

The aim of this study is to give voice to a single mother of two daughters with cognitive disabilities (Down Syndrome and delayed development), recognizing that an in-depth understanding of her coping processes will enable educational and interdisciplinary teams to get to know the needs and challenges facing mothers in this special parenting experience. Accordingly, they will be able to provide proper support, nurture collaboration and maintain them in accordance with mothers and whole families' dynamics and needs.

Research question: What is the parenting experience of a single mother of two daughters with cognitive disabilities?

## 3. Methodology

We chose to listen to the voice of a single mother (widow) bringing up two daughters with cognitive disabilities and examine her parenting experience. Giving voice to this mother is likely to illuminate day-to-day coping of mothers, who in most cases take on the majority of the burden of bringing up children with special needs. We hope that insights gained from this research will enable educational and professional teams to generate appropriate and effective support for mothers and consequently to create a platform for fruitful and effective collaborations for pupils, families and educational institutions as one.

Research method: This is a narrative study. We chose this type of research because it turns to the emotional and experiential areas that cannot be quantified [32]. It is bases on an interpretive and naturalist approach that seeks to expose the meanings that people attribute to themselves and phenomena in their world. The main role of narrative researchers is to help people to tell their stories [33]. It is not possible to recreate what really happened because people assign meaning to events always in hindsight, as a result of their consequences [34]. As such a researcher does not pretend to lay down rules or laws of reality or specific components and does not require external or internal validation [35].

Research population: Ilanit, a woman in her sixties, a widow, living in central Israel, from a low socio-economic status. Bringing up two daughters with special needs: The elder [35] with Down Syndrome and the second [30] with cognitive disabilities. Both were educated within educational frameworks until age 21. Since birth until today, they have been living at home. There has never been any attempt to move them to an external framework. Today they go out in the morning to an employment framework and return home in the afternoon.

Research tools: In this research we used a narrative interview and researchers' reflective diaries. The narrative interview was the main tool for gathering data. The diaries served as further tools to cross check the data.

The purpose of narrative interviews is to understand people's experiences and the meanings they attribute to them. In this interview there was information that was requested, but that did not come up despite this [36]. Reference to information was made with the understanding that the process of choosing details of these experiences, reflections about them and their organization, turns the stories into a process of attributing meaning. Every word used by participants in telling their stories is a microcosm of their consciousness [33].

Researchers' reflective diary: During this study, the researchers used a researcher's diary right from the start, prior to interviewing the mother and until the final stages of the work. In this diary, notes about emotions, problems, ideas and feelings, preferences and constraints were kept without filter or criticism. In addition, we raised questions and dilemmas during the work.

Research procedure: The research began with an interview that took place at Ilanit's home, on a cold winter's day. The interview took place over a period of about three hours in one sitting. Ilanit knew the purpose of the interview and told the story of her life in her preferred order. The interview was recorded and transcribed to 38 pages. In the second part of the interview, questions to clarify and expand on what she had said previously were asked. Later the interview was analyzed based on a holistic analysis according to Lieblich et al. [36] content and format analysis model. According to this model, it is possible to analyze life stories in accordance with two main dimensions: (a) Relating to the story as a whole rather than breaking it down into parts; (b) focus on the story's content rather than its form.

Ethics: The interviewee participated of her own free will. She signed a form consenting to participate in the research after receiving full information regarding its purpose, recording and transcription. In order to protect the privacy and safeguard the anonymity of the interviewee and her daughters, false names and changes to any items that may expose their identity or that of others from her story have been changed. In addition, as researchers, we based our reciprocity and collaboration with the interviewee on honesty, empathy and being non-judgmental.

Researchers' place: Rivk's acquaintance with Ilanit started about thirty years ago when she was a special education teacher and Ilanit's daughters were still young. The acquaintance has been over many years with great appreciation for Ilanit and the way she has dealt with her daughters' differences. Inbal met Ilanit for the purposes of this research as part of her studies towards her graduate degree in Special Education. We both thought that it was important to make her voice heard and she responded willingly.

## 4. Results

This chapter presents our findings after a holistic analysis of the interview with Ilanit. First, we will present her life story through milestones in her family life and afterwards we will concentrate on a profile of her two daughters as they were presented in the interview. In the second phase, we will raise three central themes from her life story: Messages, support and clothes as a mirror of the soul.

Ilanit is a pretty woman in her sixties. In the early 1980's, she married Yair, a successful businessman and they had two daughters with disabilities: The elder with Down Syndrome [35] and the second with cognitive disabilities [30]. In between these two births, a son was born who died 10 days later as a result of birth complications. In the daughters' early years, their standard of living was high. They lived in a lovely suburb in central Israel and could afford to pay for afternoon care for their daughters. Since Yair's business was overseas, “*We had an office in Rome*”, he was often absent for long periods of time. Ilanit, however, did not work and devoted her time to caring for her daughters. But this plentiful period did not last long. A few years after the birth of their younger daughter, the father, Yair, lost everything and got into serious debt. Since then, he no longer worked and died of a heart attack 14 years ago. Ilanit was forced to turn to welfare services and at the same time she began to work at times selling clothes from home. For the last ten years she has been in a new relationship, but other than one sentence she did not refer to this matter at all.

In the first part of the interview, Ilanits told the story of her life. It appears that she extracted from every story the events that met my (Inbal's) demands as an interviewer, as a graduate student in special education. She agreed to be interviewed because I was sent to her by Rivka, therefore a special connection from the past existed. She wanted to know about me, what I was studying, what I did, etc. The daughters were the primary images in her life story: 64% of her story was devoted to her elder daughter, 34% to her younger, only two lines were devoted to the story of her baby who died after the birth of her elder daughter and one line to their economic collapse.

## 5. Daughters' profile

Ilanit began her life story from the birth of her elder daughter, Sharon. She pointed out that hers was a regular birth and then she moved on to when Sharon was three months old, the age at which, in her words, health problems began to emerge. For three years she was hospitalized on and off with chronic illness. As she explained:

“She had lots and lots of shortness of breath, bronchial ahhhh... She was in an oxygen tent for three years... Because home supply was really scarce. Not now. It really was like that… We didn't operate at home... And it really was life threatening, when we got to hospital. On the way we turned to Magen David that an ambulance and all the cortisone injections, they filled her with cortisone. There was a period when she really had a face as full as the moon. And all her functions... three years that you are in an oxygen tent, and there is no... Really she would come home for a holiday, maybe for a day or two and straightaway I had to take her back to hospital. And filled with antibiotics, filled with medications…”

Ilanit described her daughter's bad state of health and emphasized that her life was constantly in danger with words such as “many” and “really”. She used many words that derived from the root “fill” in order to emphasize the number of medications her daughter was receiving. In her words, the care “saved her life”, but alongside this benefit, there was also a loss. The doctors told her that “it would damage her functioning”. Ilanit pointed to the mass of treatments as the deciding factor in her daughter's disability. In her words, “*they didn't come and say, listen you don't have a child who is developing well, as if they didn't indicate and didn't say to be that there is a diagnosis of something that wasn't right, do you understand?*” The baby's physical development was sound, “*her whole body was small, and her face was so big... You would not believe that under the blanket was a body so small and as if it wasn't... Developing*”.

The difficult period of hospitalizations ended approximately at the age of three and at the age of four, Ilanit placed Sharon into an educational framework for the first time, into a Montessori kindergarten. A framework that Ilanit praised a number of times. Then Sharon went into first grade, in a special education class. During the afternoons, Sharon received enrichment in many areas: Occupational therapy, speech therapy, a private teacher to teach reading and writing skills, art and gym classes. When this elementary school closed, Sharon was transferred in the third grade to a special education school for children with light to medium developmental delays called “Brosh”. At age 12, Ilanit transferred Sharon to “Yellin” school, because, in her words, “*It was considered, as if, I always knew that it was considered the best school, but it really was the best*”.

For the remainder of the interview, Ilanit focused on her daughter Sharon's abilities. “*She knows a lot, look she knows the news, she knows what is going on in the world, she knows gossip. She reads ‘LaIsha’ (a weekly woman's magazine), she reads, she hears as if she is not detached*”. Ilanit employed many verbs in order to emphasize her daughter's abilities and used the verb “know” four times in one sentence in order to hone in on this idea. Later she even emphasized her memory “*and her memory… 20 years later she will tell me (names of people whom she passed in the street)... she's also the only one who remembered the names of all the children...*” Ilanit described Sharon's love of expensive jewelry and related her knowledge and “*expensive taste*” to the fact that she used to go around with her during the period of plenty. Alongside aesthetics, she described Sharon as a “*little fatty*” because her thyroid was depressed and the having to take cortisone. She used maternal expressions as a way of delicately expressing that her daughter was whole.

Her second daughter, Hodaya, was born when Sharon was almost five years old. Ilanit told me, “*when she was born, everything was okay. After about a month and a bit, suddenly she had a very high fever and started to vomit*”. Ilanit told about a sudden turn in her baby's state of health and described it in detail:

“*Fever, vomiting, okay, go to the doctor... A week passes, everything is okay, another week and she again has a high fever and is vomiting. Go to the doctor again... In Israel they start doing tests and it's all so slow, and everything is okay. But the child is fine one week and vomiting the next. Even now, if at the beginning you vomit... every hour or two hours, everything is not okay, but at its height, she would eat, vomit, eat, vomit*”.

It appears that the use of so many verbs and the words “again” and “okay/fine” was meant to emphasize the unexplained loop in which Hodaya was found—a vicious circle between sound health and periods of vomiting. In order to maintain her body weight, they would drizzle a special substance from the U.S. into her stomach, using a gastro-nasal tube. “*There were days when I was going to the hospital every other day. In order to save a few grams.*” Ilanit used the word fought in order to describe their struggle to keep weight on their daughter “*really we fought for every gram in a... certain period*”. This is the only time she used this expression with a combative manner.

Ilanit said “*the little one was something so rare in the world*”. As a consequence of their initiative, they were invited to Mount Sinai, one of the oldest, largest and best hospitals in the U.S.A. that specializes in medical genetics, pregnancy and birth, with a wide range of children's departments including a research institute. They stayed there for about a year and a half, “*a doctor came from Canada, another from China, another from South Africa and one woman docto, as if she had an Ashkenazi genetic problem, as if to negate it*”. Despite the doctors' efforts, they came up with nothing, “*they didn't find anything. So there wasn't even a diagnosis, they didn't know what it was, there was nothing, nothing*”. It appears that the use of negatives five times hints at the frustration that Ilanit felt when nothing was diagnosed. After they returned from the U.S.A., her husband travelled to an “*institute in Rotterdam, Holland*”. In her words this was “*the best genetic institute in the world*”.

Even on her return from the U.S.A., Ilanit continued to spend periods of time at hospital. At age one and a half, Hodaya still did not smile, “*as if her eyes were already more awake, as if at a year and a half... but ah... as if there was no reaction... I don't remember if she ever smiled*”. Attempts to get her daughter to gain weight did not bear fruit. “*She reached the age of three at a low weight. She weighed 2.7 kg at birth... and that is what she was at aged three*”. At the age of approximately five, she entered an AKIM^1^ kindergarten. Everyone was already standing, but she was the only one rolling on the floor in order to move in space, “*she wasn't walking yet, wasn't standing, wasn't walking*”. Ilanit told me that they used to go to Eilat in order to let her swim and develop motor skills. “*We'd spend whole day with her in the water. As if the water would help her.*” She spoke yearningly about their visits to Eilat, “*It was a lovely period. We always remember that period as very lovely*”.

The more Hodaya developed, the more her difficulties in areas of learning became noticeable, “*Reading and writing was really difficult for her. As if... she did not... really manage to learn.*” In contrast, she showed a great interest in the area of computerization, “*You wouldn't know how to make it work, she would look at it and know how to work it. She was not afraid, you know, to press buttons*”. Hodaya studied at “Brosh” school from Grade 1 to age 21.

When the girls finished school at age 21, they remained at home without a framework for about a year and a half. Thereafter, attempts were made to integrated them into permanent employment through “MAAS” (Rehabilitation Employment for People with Developmental Delays), a framework that integrates training and rehabilitation with places of work. Sharon worked in a cafe, but when that closed she worked in a hotel and afterwards for someone in a private home. Today she works outside a “MAAS” center for three hours per day. Hodaya, in contrast, still has not managed to integrate into work outside this framework, “*She knows exactly where she has to go, at what time, she sits, she doesn't have a wristwatch... now this really disturbs the group leaders*”. In the last year and a half, problems have arisen in Hodaya's functioning. The team asked Ilanit to take her to a psychiatrist and as a consequence she now has to take tranquilizers. Lately the situation has worsened and Ilanit has turned to a behavioral advisor in order to help Hodaya. The advisor told Ilanit, “*I thought that this was the easiest case, but it is the most difficult. He is not getting anywhere with her*”.

In conclusion, Sharon and Hodaya were born five years apart, Sharon with Down Syndrome and Hodaya with cognitive disabilities. Both had unusual health problems soon after birth. They were educated in a special education framework from a young age. Sharon successfully acquired reading fundamentals and it appears that she has a higher functioning level than Hodaya. Once they had completed their educational framework, they began to work in protected employment near to their place of residence. It appears that Sharon integrated well in contrast to Hodaya.

The subjects that emerged from Ilanit's life story were rearranged according to central themes as follows:

1. Messages: Observations through a glass half full, to refer to the girls as normal, not without my daughters
2. Support—internal circle, external circle
3. Clothes as a mirror of the soul

Each theme was analyzed by interpretive content and form analysis.

(1) Messages

Throughout the interview, Ilanit raised a number of messages and observations about life. Sometimes she said them explicitly, while at others they were derived from her words, sentences and issues themselves.

“I believed it would be good”

The central message in Ilanit's life story is to see the glass as half full and continue onwards. She did not say this explicitly, but it was threaded throughout the interview. In contrast, dramatic events that belong to a half empty glass, were not recalled at all or alternatively were mentioned briefly and without much weight.

When Ilanit began her life story she described her parenting experience when Sharon was already three months old. She skipped over the birth experience and did not at all refer to the turning point in her life when they were advised that their daughter had Down Syndrome (researcher's diary). Such a situation disrupts family balance and challenges any new parents. But later she said in one sentence, “*Listen, all of a sudden your whole life turns upside down*”. This sentence took on a significant weight in relation to the spirit of this interview, a kind of roaring silence. It was the first and last time that she mentioned this. Even when describing the birth of her younger daughter, she said nothing about moments of crisis with regard to the addition of another child to the family unit, and how much more so when referring to another daughter with cognitive disabilities.

During the periods of both her daughters' infancies, she brought the health problems to the forefront and described them in detail. When she was asked how a young couple managed to deal with the situation, she answered, “*You don't have time even to think. You have no time to think because you are so*...” This sentence was repeated a number of times in this context. Repetition emphasizes the fact that Ilanit chose to focus on doing and devoted care of her daughter and invested less in thinking about the situation. Even if she had crises moments, she chose to broadcast it differently, “*Listen, you sit, what can I tell you, day and night (at hospital)... let's say I didn't broadcast it...*”

Ilanit testified about herself that she focuses on the present, “*what will be, nothing will be*...” and declared that she was always optimistic, “*always... first of all, I believed it would... be good*”. However, she immediately restricted her words and said, “*that's the idiotic bit. Do you understand? But because of this I'm a blonde, I'm allowed*”. It appears that she understood just how much reality challenged her and despite this chose to see her glass as half full. Ilanit mocked herself with a wink, but it's safe to assume that this approach helped her cope with life's vicissitudes. Today, through a mirror of time, she understood just how optimistic she was, “*I believed it would be okay. Why the idiotic part?... Today I see my niece, let her be healthy, with a two-year old daughter and it's really something that... as if I didn't experience it with the girls*”. Close observation of her niece's daughter's healthy development made Ilanit think about the differences between her daughters' development and normal development.

Between the births of her two daughters, another baby was born who died “*My placenta burst, and he died after 10 days*”. Ilanit did not describe the fears that accompanied her during her second pregnancy, expecting a son, the shock when he died and coping with this tragedy. In her words, his death was caused by a “*hospital mistake*”. She did not add any details and even signed off on the description with one sentence, “*but it can't be helped, it happened*”. She left the tragedy behind and continued forward.

Likewise, she did not go into detail about the period when her late husband lost all his assets, a situation that she has not yet managed to overcome. She indicated with regard to investing in her younger daughter, “*and then we also had an economic fall, so then we couldn't invest like we had with the elder one*’. Despite her low economic status, she chose to stand tall literally, “*Now it's true, a person who doesn't have money, sits all bent over and all that. I at that same moment... and this is what happens to me, I have moments that they are a little psycho... I sat up straight (smiles and sits up straight)... there is no one like me*”. Here too, just like the above description, she criticized herself with a smile and humor, by sitting up straight despite the given situation (low economic status).

Towards the end of the interview another aspect of Ilanit's life emerged and that was her belief in God. Ilanit, who grew up in a traditional home, said that over the years (when the girls were aged 12 and 8 approximately), she began to attend lectures given by Rabbis and later even described herself as “*being at home*” at Rabbi Kanievsky's home in Bnei Barak. She repeated this expression time and again (at least 6 times) with regard to the subject. Even her late husband began to go to lectures, in her footsteps, “*Rabbi... knew my husband well*”. These visits did not lead them to become newly religious, but it seems that closeness to religion opened another horizon for them, that helped them to cope with their reality and clarify their observations of a half-filled glass.

“*I once said to someone thank God... thank God that there are situations that are a million times worse, that I see. Yes? When I go to MAAS, when I go to school, it's as if you go to a place where you see them all together (all those who are disabled), then the girls generally, there is nothing, there is nothing like that, I'm not just saying it... I don't argue with Him (God). Aah... Don't argue with Him, on the contrary. Once someone else said to me, you still believe in God after all ah... I always say thanks very much to God. Because firstly, they're wonderful and... I told you, I always look at the part that really could have been much worse... so you see such cases that all you can say is give me strength*”.

The special education framework introduced Ilanit to others with disabilities and as such she was able to compare them to her daughters and understand that it could have been worse. She “*did not argue with God*” and even thanked him from the bottom of her heart. She used the work “thank” that they were her daughters over and over again. When I said to Ilanit, “*surely, it difficult for you*...” she stopped me and explained to me why the word “difficult” did not exist in the lexicon of her life.

*“I saw it isn't easy. Yes? Not difficult. I don't use the word difficult because it can always be more difficult. You know, when God says, if you say difficult, then He will show you what difficult is. Say easy and then He will make it even easier.* *Because, you see, there is no limit to difficult. It's as if I say to you that I'm coming... to places of work and that I always see children at school.* *You know, that God will care. So really there are easier cases, but there are those that are much more difficult*”.

Ilanit adopted a vocabulary that semantically matched her core message. The message is entwined in her life story as a sort of pillar of fire to light her way, and it is safe to assume that it helped her cope with bringing up her daughters.

I must point out that towards the end of the interview a crack was revealed in the half full glass, at the point when she was asked about her professional development. As a consequence of the birth of her first daughter, Ilanit stopped working and the feeling that she missed out is with her to this day, “*You don't develop in the directions that you want. You don't do what you want. It's not... if I tell you that I'm something that I it... it no! I could have, could have, could have done a million more things*”. Repeated use of negative words and the verb “could have” emphasized the frustration that Ilanit felt in not being able to realize the “millions” of things that she could have done if things had been different. It was not for nothing that she chose a sparrow as a symbol of freedom and release. Even when her husband opened a business for her and she felt like she was succeeding, he asked her to close it, “*I didn't marry a woman who would support me*”. It seems that her need to work was part of her desire to be free from her busy daily life. Like a bird held in a golden cage, she did not fulfill herself.

When her husband, Yair, lost his assets, and later when he died, and the gold dissipated, her life became more complex. Not only did she have to cope with bringing up her daughters, but without a husband or her mother who had died, she had to worry about making a living. She started selling clothes privately on Friday nights, “*I started doing this from home... and I could have achieved a lot, lot, lot, lot, lo(t)... succeed very greatly*”. Ilanit used the word “lot” five times in order to the emphasize the potential hidden in the business she had opened, “*See world, see this, you meet people, you do, you work, you do a thousand and one things that you can that... simply I neutralized everything*”. The thing that burst her fantasy, that did not allow her to take off was the fact that she had two daughters with disabilities, “*I couldn't do the business and travel and do what I wanted. They were both at home. You can't. Not work, not dream about working. Nothing*”. It appears that the conflict between motherhood and self-fulfillment has not yet been resolved for Ilanit. She acknowledges her abilities and feels that something big is missing from her life from a professional development aspect.

“I don't relate to them as different children”

Ilanit stated this explicitly and throughout the interview, one could grasp this theme with regard to the way she had chosen to bring up her daughters, “*every child has character from the age of one week, therefore one has to respect them... first of all as human beings*”. She described her efforts to have her daughters mix with normal children during their childhood:

“*I fed a whole neighborhood who were interested... all the normal girls, I would call them to come and eat... because it was good for my girls. As if they didn't have a neighborhood where... children ahem... who were different, you know what I mean. So, then I said that I fed a whole neighborhood of children, so what? I* *wanted to or not. I did what was good for the girls, that's what I felt was good for them*”.

Ilanit chose to bring her daughters closer to normal girls through giving. Despite the fact that she did not want to, she operated out of a desire to do what was good for her daughters. Even when her daughters grew up, she provided them with the same expertise and life skills that every adolescent entering the world of adulthood gets.

“*As child aged 0 you have to learn them. If you eat one pita, it does not mean that you eat two pitas. How do sit at a table... or about their menstrual periods. I didn't wait for the school nurse to teach them.* *As if you don't teach... how many times have I said to other mothers, even take your girls into the bath with you... as if. No, no matter how much you try to explain, but it's like something that everyone interprets differently. So, I was always with them*, even arguments, in inverted commas. *Just an example, I would go with them to their swimming group... now when they got out of the bath, I would say to the teachers, why do they have to get out without a towel around them... hair under their armpits, their legs ou... as if, there are minimal things... there are things that are a, b, c*”.

She did not wait for any external factor before instilling in her daughter's hygiene and aesthetic rules in adolescence. Ilanit's theme of relating to them as normal sometimes created tension between herself and other parents. In the social sphere as well, in relationship between men and women, Ilanit believed that her elder daughter would be able to handle being one of a couple, “*She talks to me, she very much wants someone, and she would be able to do that*”. But Sharon does not want someone different.

“Today if you were to ask her what she wants, she badly wants a boyfriend and badly wants to get married, and she wants it... but she doesn't want those from her environment... sorry that... all those who are different... she wants someone with a car, who won't have, that will take her that... you understand what I mean?”

Sharon's desire is to find a partner. Even when they wanted to introduce her to someone religious, she did not agree, “*I said to her, listen he goes to synagogue, he is religious, you want to be religious, she doesn't want a religious person*...” It is reasonable to assume that her desire for a normal partner derives from Ilanit's theme that has been instilled in her over the years.

It is important to point out that the mother's message to behave as if her daughters were normal, was not always accepted by society. Ilanit chose to recall one incident in which she felt others were looking at her elder daughter because she is different. “*Sometimes **I** would see from the side, say mothers communicating with one another as if they knew the girl was different... I wouldn't move... listen carefully I wouldn't move, I wouldn't flutter an eyelid*”. Despite society's relationship with the girl's difference, Ilanit chose to ignore it. Ilanit did not describe any other similar experiences. In contrast she praised the ultra-orthodox community in this regard.

“*Imagine as if the daughters (of the Rabbi) always came and play with them. It's not something I saw amongst... the others (secular people). You know what I mean? I never saw it. Always the opposite. Whenever I went there, they would always play with them, they would take them, walk with them. As if I would feel that there weren't any partitions*”.

The partitions that Ilanit felt in secular society did not exist in the ultra-orthodox community. Even in conversations with the Rabbi, she emphasized again, “*There are no partitions... its person to person*”. They even accepted her unconditionally, “*He (the Rabbi) accepted me as I am*”. Even after her husband had died, she told the Rabbi that she has a boyfriend, “*I told him**:** I now have a boyfriend. I spent the festival with him*” and the Rabbi respected this and was happy for her.

It is interesting to note that throughout the interviews she did not use the definition “professional” about her daughter's disabilities. I am of the opinion that the medical team informed the parents of their daughter's syndrome (researcher's diary) particularly because of the fact that her disability was visible. With regard to the younger daughter too she said that she was “*a very rare case worldwide*” but there are “*no definitions*” and this was after their return from a research institute in the U.S.A. “*Listen, to an extent you are pleased that they didn't find anything*”. It appears that her choice to relate to her daughters as normal made definitions irrelevant to her life story. It is possible that this too is the reason why Ilanit only participated once in a placement and other committees, “*I sent my husband*”. It appears that her difficulty in confronting a different reality prevented her from attending these types of meetings and as such allowed her to build a protective wall against all formal definitions of her daughters' disabilities. In one of her conversations with doctors she declared, “*I told him: Listen, I want to be like everyone else*”. Therefore, her relationship with her daughter is firstly as human beings.

“Not without my daughters”

Another of Ilanit's themes that is a recurring theme in her interviews is that she would never give up her daughters. When Ilanit was asked to give a name to her life story, she chose one that emphasized this theme, “Not without my daughters” (researcher's diary). They are part of her and they accompany her everywhere. She has always gone out with them shopping and for entertainment, events and trips both in Israel and abroad.

This perception that they are part of her and that she is not prepared to put them in institutions, started when they were infants, “*My private doctor, who was the director said to me one day, lets hospitalize her in an institution. From that moment onwards, he was no longer my doctor either. As if he knew that wasn't what was in my mind and nothing*”. Ilanit chose to cut herself off from this doctor that dared to raise the issue and speak about putting her daughter in an institution. Even today, when her daughters are in their thirties, Ilanit hesitates to think about a hostel and would consider the idea only if she found a perfect place, “*If tomorrow I saw that there was a place, some wonderful framework and if I thought that it was absolutely perfect, and there is no perfection*”. She has set the bar high knowing that she will not find a perfect hostel. It seems that Ilanit is conflicted between the desire to keep her daughters close to her and an understanding that a hostel framework “*maybe would really be better... No one knows what tomorrow will bring... (longer than usual silence)... it would be right to really do, to take them out*”. Despite her understanding and admission that it would be better for them, she is not prepared to give them up. When she was asked about the future, she said that she did not think about the future, “*I'm a blonde, it's an advantage. You understand?*” Her choice not to think about the future and investigate the options her daughter have preserved Ilanit's position of ignorance. Here too, as before, Ilanit chose cynical humor, “*I'm a blonde*” in order to explain unresolved issues.

As part of her daily concern, Ilanit is punctilious about being at home when her daughters finish work. She stated more than five times that she does not leave them alone during the afternoon, even if it damages her social conduct.

“*A lovely girl came. But she doesn't understand that I don't have time for her... as if, I won't leave the girls in the afternoon and go to her. You understand? As if her needs are different and my needs are different... and many friends of that age, yes, their children are no longer at home. Do you understand? They sometimes have to babysit for their grandchildren and sometimes do something else. It's... but they have more free time... I won't go with you to sit in a cafe on Shabbat and nice, they will be at home alone. As if for my part, do you want me after eight, after nine after ten at night, great!*”

In contrast to her friends who are available in the afternoons and take part in routine grandparental activities, Ilanit does not have this privilege. It is important for her to be at home when her daughters return from work (MAAS). She allows them to carry out tasks independently during afternoon hours, such as: Buying certain items, shopping at Superpharm, but “*they know that I'm at home, I'm waiting for them*”. Despite the constraints, Ilanit has not given up on a social life. During the interview a number of friends telephoned and from what was said, it appears that she does not forego quality time and enjoyment with her friends.

One can understand perhaps the conflict in which Ilanit finds herself in light of what being a parent is to her.

“*My late mother used to say, when you have parents you are still a child. Yes? You're not on your own. As if you always know that you have, even if something is missing, your mother will always give you. Right? If a feeling that you are going, of something on which you can lean. Yes? In childhood... I say, all the time that I'm here until 120. So ahh... I try to give them the... the best there can be*.”

As long as Ilanit is alive and able to do so, she is prepared to continue bringing up her daughters. In conclusion, during the interview a number of themes recurred throughout her life story. To see a glass as half full, to relate to her daughters as normal and to keep them close to her as long as she is able.

(2) Support

Ilanit was the central figure in bringing up her daughters. Nonetheless, her close family helped her to do so. There were also supporting factors in her external circle such as educational and welfare systems. Accordingly, there are two themes: Internal support and external support.

Internal support

The father—“*I knew he was the breadwinner, so I took more upon myself*”

The father's role bringing up the girls was “*behind the scenes*”. He supported his family well but was away from home for days at a time. Ilanit took the reins of bringing up her daughters, “*I knew he was the breadwinner, so I took more upon myself*”. However, during the times her elder daughter was hospitalized, it appears that “*my husband, may his memory be blessed, I think that with the elder one at the beginning he was there too*”. But afterwards he did not take an active role “*afterwards... I spent 8 years sleeping on a fold-up chair at the hospital*”. That is the reason Ilanit spoke in the first person singular, “*I slept*”, not we slept. The turning point was when he sat at home after his economic decline. Ilanit pointed out that at first “*he helped me a lot... as if he was present at home. Listen when there is some else present... then it's more... it's a partner in life*”. The late Yair earned the title “*partner in life*” only during this period. Using this expression did not leave any doubt as to her feelings of relief that she felt with regard to bringing up her daughters.

The grandmother—“*my mother was with me”*

In contrast to the father, the grandmother (Ilanit's mother) had a significant role in the mother's dealing with bringing up her two daughters. The grandmother was loyal and responsible. Ilanit often mentioned the fact that her mother never complained, even when she had to leave work and relieve her at the hospital and return to work the next day. Thanks to her mother's help, Ilanit was able to maintain a balance over the years that she had to go to and from hospitals as well as in the years after. She managed to find time for herself to be refreshed and return with renewed powers.

“*She (the elder daughter) would come home for a couple of days, my mother would come to me... I would go out and enjoy myself immediately... suddenly there was a break of some sort, I would say to my mother that I was going crazy, I'm going away for three or four days, as if (to friends)...* *we (Ilanit and her husband) went away for the first time (when her elder daughter was aged two and a half) when we felt that the situation was better.* *And she had a very bad attack. But my mother was with her and the doctor too.... My late mother was always with me (regarding trips to Eilat)*”.

The grandmother's presence gave Ilanit some peace, and allowed her to be free, for short periods, of her responsibilities and to step out of her routine. Yair also felt this about his mother-in-law, “*He knew that my mother was at home. He didn't have to worry about anything... the girls were taken care of, everything was sorted*”. At a certain point, the grandmother went to live with Ilanit, Yair and the girls and ten years later she died of a cardiac arrest. Ilanit described her daughters' reactions, “*It was very hard for them... suddenly to be left as well... she was with me. She lived with me*”. She repeated “*she was with me, lived with me*” over and over again in order to emphasize the support she had received from her mother.

Brothers and sisters—“*my brother will be with them; my sister will also be with them*”

Aside from the grandmother, Ilanit's brother and sister were also part of her support circle. They were often woven into experiences that testified to their special connection with the girls. “*My brother, who works in computers and understands. He would sit next to her and simply did not know how she would enter a specific game that she wanted*”. The sister would go on their trips with them, “*It's nearly two years since we were in Rome with my sister Tami. They visited Paris, the elder one has been, I think, twice or three times*”. The sister is mentioned in overseas trips, trips to Eilat and even routine activities such as supermarket shopping. Today still, they help Ilanit despite the fact that they are grandparents to their own grandchildren and their time is limited, “*Now, I'm going away. So, my brother will be with them, my sister will be with them. They don't suffer... my brother is coming, he will sleep over*”. The fact that her brother will care for the girls whilst she is away, testifies to the quality of their connection. This support allowed Ilanit to maintain her sanity and even develop a relationship with her partner in the last ten years.

External support “I am anti the system”

Education system—“*Whether the girls went to school or not... it's the same thing*”

Right at the start of the interview Ilanit pointed out, “*You represent the system and I am anti the system*”. This saying testifies to her perception with regard to systems, whatever they are. Of all the frameworks that belong to “the system”, Ilanit expanded greatly on the Montessori kindergarten, with which she was very satisfied and on the other hand, the Hodaya's employment framework with whose conduct she is dissatisfied.

The Montessori kindergarten provided her daughter Sharon with the best “*there was a pool and petting zoo, and there she really got a... that kindergarten gave her so very much*”. Later she said, “*it was the only one then, with a swimming pool where they would teach, they would teach children English there... it was a large staff team, there were also lots of children... she got like... a lot there, a lot*”. It seems that the abundance of activities and large team turned the kindergarten into a well appreciated one for Ilanit, as evidenced by the number of times she used the words “a lot”. In addition to everything that Sharon got during her time there, they also funded treatments in the afternoon, “*In the afternoons she would be given speech therapy and occupational therapy, everything, everything, everything privately*”. Ilanit repeated the word “everything” three times, to emphasize the huge enrichment that Sharon received.

When Sharon moved to Grades 1–2, she learned how to read quickly. According to Ilanit, she achieved this thanks to their economic status that enabled them to fund groups and other reinforcements, “*everything privately. That means, that from my point of view, if the child had gone to school or not gone to school, it would have been the same. Because I gave her all the groups in the afternoon*”. From what she said, their belonging to the system was, it seems, against her will. If she could have kept Sharon at home, she would have done so.

Welfare—“*if you want something, then there is no budget*”

Support from the welfare system did not have satisfactory results from Ilanit's point of view for a number of reasons. Firstly, lack of knowledge with regard to things for which she is eligible, “*And I went to the social worker and I told her that I want her to tell me what I am entitled to. What, you need a bed? I told her that I did not need a bed. I needed her to tell me what I was entitled to. That's also something that you never hear anything about. That's concealing information*”. Secondly, according to her, social workers change at a very fast rate, “*They are all young and here now I also have a new one and last week, I was with who... in six months' time there will be a new social worker. Come let's meet her, let's sit and talk*”.

While she was forced to rely on the assistance of external support factors, Ilanit understood that if she did not help herself, no one else would help her. She said this explicitly when telling about the time she wanted to share another mother in her insights, “*The young one began to say: I want, and I want and I want... I got up and said: I'm sorry that I'm disturbing you! I just have to tell you one thing... but you will learn there that you will have to do for your children alone. You won't get anything of what she wants and wants and wants and wants*”. Ilanit learned her way around the system and understood that support was at the most basic level. If she did not help herself, salvation would not come for a systemic source, and therefore she no longer asks anything of the welfare system, “*Now, how do they say it to me, you don't ask for anything. So what, I also have nothing to ask of you and even if you want something, there is no budget*”.

In conclusion, Ilanit declared herself as against the system. Through her words and the issue she raised, one can feel it. Despite this, there were frameworks that provided good experiences for Ilanit despite the fact that her relationship with them were ambivalent.

Employment frameworks—“*the staff are tired sometimes, it is very hard work*”

Ilanit pointed out another framework, that of her daughters' employment and particularly Hodaya's in light of her integration problems, “*for more than a year someone told me that the younger one needs a psychiatrist now I am very against*”. Ilanit went to the psychiatrist and he recommended tranquilizers, “*Give her Calmanervin to calm the staff*”. The semantics here emphasized Ilana's lack of faith in the staff. Medication to calm the staff and not Hodaya. In the same week as the interview, Ilanit was told that the situation was getting worse. She turned to a behavioral adviser, but that also did not result in anything. The private sector also disappointed Ilanit, “*from my point of view, I pay him money I know that it's also a wasted effort*”. Ilanit turned to a second psychiatrist that the behavioral adviser recommended. In this interaction too, Ilanit explained that the problem was with the educational staff and not her daughter, “*I told her to listen well, the staff are sometimes tired, it is very hard work, very exhausting... there are some who come to work for MAAS and in the hostels and not always do they have the right approach or have learned about these things*”. Ilanit did not agree to medicinal treatment and did not succumb to the pressure exerted on her by the system.

“*Which of them they want me to give them, and she said to me, do you have the strength to deal with the system and I said that I do! I won't give her any tablet that might harm other things with her and I am against it. She is not in a state that I have to give ahh... to give it to her. Their responsibility is the responsibility ahh... when they need, she comes to work*”.

Alongside her complaints about MAAS, Ilanit acknowledges, “*nothing will come out of her that will enable her to work outside or something like that*” and even added praise, “*they give her a lot of warmth, there is yoga and judo and art and therapy, they invest a lot in MAAS*”.

(3) Clothes as a window to the soul and perception of life—“*you already dress in another way... it doesn't interest you*”

Throughout her life story, clothes and aesthetics are a constant thread in different times and situations in Ilanit's life. The deeper I went into the interview, the more I understood that clothing expressed a mirror into Ilanit's soul, as a symbol of mourning, happiness, as a way of demonstrating her messages, both consciously and subconsciously. This is what she said about her eldest daughter's hospitalization periods:

“*I came to the hospital in the same clothes I had worn the day before. But my late husband said, she's going crazy how do you come like this, day after day with the same... slippers already, you don't take an interest. As if saying, not that it doesn't interest you*”.

Perhaps Ilanit “did not broadcast”, in her words, what she went through, but clothing revealed her feelings. As a well-dressed woman punctilious about her appearance, arriving in a public place in the same clothes and slippers is perceived by her husband as if she had “gone crazy”. It appears that like a person in distress, nothing interests her anymore. Later she returned to this subject and testified about herself, “*All my life now is hospital. What... hospital. You already dress differently than as if you feel that life is not exactly*...”

Throughout the interview Ilanit described again and again her daughters' activities around how one looks, “*and the elder one, if her blow dried hair doesn't stay in place... she wants to go to the hairdresser every day and a manicure and a pedicure. Like that... as if she has to. Do you understand? But now the little one... give her any new clothes*”. It seems that the girls care for their external appearance was influenced to some extent by their upbringing and how they have been nurtured from childhood. Ilanit declared openly and without hesitation that it was important to her that the girls were aesthetic in their appearance, “*If I... if they go out with me in the evening or go with me somewhere, I won't give them something that I don't think suits them*...” She pointed out that she did not care about others' reactions but in the same breath she said, “*most people what (will they say)... look at the mother, what does she care about her daughters, how they look how is that?*” It seems that her insistence that the girls dress aesthetically was a result of Ilanit's message throughout her life story, to relate to the girls as normal. “*That they are different and this development, they are not idiots (silence)... they are not idiots. They know exactly... they want to go to the hairdresser. And if you use permanent make up, they also want permanent makeup*”. That is to say, their difference will not prevent them from striving for an acceptable model of beauty. It seems that busying themselves with a fitting appearance and aesthetics, comprises cover and compensation for their looks that hint at their disabilities. Even when turning to a social worker, Ilanit told her “*they need more from you to get dressed*”. In other words, because of their disabilities, they have to take more care to appear suitable, why? In order to hide that they are disabled? Is that so?

Their economic downfall and reduction in their standard of living over the years did not prevent Ilanit from continuing to look after herself and her daughters. According to Ilanit, looking good and nurturing the girls, created an illusion that everything was fine from an economic point of view and this was an obstacle with regard to other school parents. At one of the parents' meetings at “Yellin”, she arrived looking like a model, despite the fact that “*we had nothing to eat... we didn't even have money to pay the class committee*”. One of the mothers turned to her and said, “*Tell me, I heard that you didn't pay the committee*...” Ilanit explained “*I even dye my hair by myself, when I say I don't have, I don't have*”. Ilanit and her daughters' punctilious appearance create a dissonance between their external appearance and their statement that they have no money.

Even with regard to the welfare system, her clothing was an obstacle, “*You go to see the social worker. What did she say to me? I see that she is well dressed... These remarks, I don't like them*”. Since then Ilanit decided to dress simply when she goes to a National Insurance office, “*When I go to a National Insurance office, I don't put on makeup. I go with a... what can I tell you... this blouse is thirty years old... dddd doesn't matter. I say that it is always for luck*”. She attributes magical powers of luck to simple dress.

Unexpectedly, crossing to the ultra-orthodox world has not caused Ilanit to change her appearance. The opposite, despite the clear laws and rules with regard to modest dress, she has maintained her appearance, “*I would also go to the lessons, also sometimes... in shorts. I would come, sit down next to the Rabbi, put the tablecloth like this*”. Even when she went in modest clothing, the Rabbi said to her, “*Are you in disguise?!*” This conduct reinforces Ilanit's feeling that the ultra-orthodox world accepted her and her daughters unconditionally. It is not for nothing that she feels “at home”, an expression she repeated a number of times.

In conclusion, when examining the profile of the daughters and the key themes that arose, including secondary themes, it is possible to conclude that Ilanit adapted for herself different coping mechanisms in her role as parent to two daughters with disabilities. Indeed, at the start, one can feel her low emotional state, when she understood that her life had turned upside down. But quickly, she focused on doing and devoted care of her daughters. It seems that the gap between an expected child and reality was not closed but exchanged for a clear message “*to relate to the girls as normal*” and as such, ignoring medical definitions. Another means of coping with crises and challenges was thanks to her optimistic personality that allowed her to adopt the motto of seeing a glass half full. Ilanit maintained a balance between caring for and nurturing her daughters and getting out of the routine (such as: Enjoying herself with friends, travelling in Israel and abroad with the girls or without them) and as such managed to keep her “sanity” and refresh her spiritual strengths. This would not have been possible without the support that she received from her close family: Her late mother, her brother and sister. In addition, turning to religion was another way to experience parenthood from a more spiritual angle.

It should be noted that dress and aesthetics decorated her life story from when she was single (which was not recorded at her request), during times of success and the downturn that came thereafter, from the birth of her daughter to today. On the face of things, dealing with trifles, but going deeper into her life story raises a reasonable explanation for her desire to remain well cared for and to care for her daughters is another type of coping mechanism, a type of compensation and a means of erasing or softening challenges with which she has to deal.

## 6. Discussion and conclusions

In this research, it was found that the parenting experience of a single mother to two daughters with cognitive disabilities is complex and filled with challenges. This special type of parenthood is influenced by different factors such as: Values, beliefs, economic status etc. The mother's internal resources and family support were also significant factors in the process of constructing this parenthood.

According to the research literature, the birth of a child with cognitive disabilities creates a different functioning system than usual, and consequently physical load, feelings of shame and failure, self-blame and more [14]. This information was requested and yet did not arise explicitly in her story. It seems that the point in time at which Ilanit was interviewed, affected the way in which she chose to tell her story. Nevertheless, observation under a microscopic lens reveals remnants of feelings of depression and stress, evident of emotion focused coping. Between never ending descriptions of positive coping, Ilanit said, “*Suddenly my life turned upside down*”, a sentence that was very significant in comparison to the spirit of the rest of her life story. Low spirits arose as well in the central theme about clothing as a mirror of the soul. As a woman to whom external appearance and aesthetics are import, arriving at hospital wearing the same clothes testified to a state of distress.

Alongside emotion focused coping, action focused coping appeared simultaneously, whose main aim was to try and improve the existing situation. The mother adapted quickly to taking devoted care of her daughter with chronic health problems, regardless of the severity of her condition [11]. After a number of years, she was forced to go through the same cycle of hospitalizations, when her second daughter was born. After this sage of hospitalizations, the desire to provide her daughters with any treatment that could advance them was created. Thanks to their economic status at that time, the girls received unlimited treatments. The mother even went as far as to spend a year and a half at a genetic institute in the United States in order to find answers to the rarity of her daughter's illness.

It appears that the tensions and distress that were hinted at in their early childhood years, lessened in their later childhood when the girls stabilized, from a medical point of view, and entered formal frameworks [14]. However, life challenged her enormously without any connection to complicated parenthood. The death of a baby, economic fall, early death of her husband and more created a complicated reality alongside special parenthood. Despite this, Ilanit chose to see the glass as half full, a central theme that flowed through her life story. Her optimism allowed her to maintain a balance and deal with unexpected situations [22].

As the year passed and childhood years were distanced from the mother's consciousness, it appears that she underwent an internal change that led her to insights that influenced her ways of coping. She chose to renounce descriptions that labeled her daughters disabilities. As a consequence of this insight, she stated that it was her desire to experience parenthood “*like anyone else*” and chose to relate to her daughters “*as human beings*”. It appears that this is the reason why she did not attend placement committee meetings, she operated according to her feelings and listened to her inner voice. These committees, for her, formally perpetuated her daughters' disabilities and therefore she chose to do away with them, just as she chose to fire the doctor that suggested putting her daughter (during childhood) into care outside the home.

It appears that this message marked her way and influenced meanings that she gave to different events in hindsight [34]. For example, memories of her daughters' birth and infancy were shown in a different light. Her daughters' health problems moved to front of stage and became the central factor that led to their disabilities, as indeed in her words, “*they were born normal*” and only after a month were any problems uncovered. Dealing with chronic illness is no less complicated that dealing with bringing up children with special needs [10], but this “preference” derived, in my humble opinion, from a choice to soften feelings of guilt and mourning, that often accompany parents of children with special needs. In addition, in this way she diverted reasons for her daughters' disabilities to an external factor, the hand of fate.

In contrast, apparently the mother did not allow the hand of fate to interfere with the level of her daughters' differences. How can that be? She often compared her daughters to other different children whose cases were much harder. This comparison created a feeling of control. Indeed, if it was possible to choose, it is clear that she would have chosen the situation in which she finds herself today in contrast to motherhood of “*all the (other) different people*”. This thought reduced stress because she structured the situation as if it was a chosen output and not something that was dictated from above [19].

Alongside the desire to relate to her daughters “*as human beings*”, Ilanit was forced to deal with their external difference (researcher's diary), another dimension that makes it difficult for parents of children with special needs to cope, when their difference is visible [3]. In her unique way, she managed to deal with this fact by being over punctilious about her daughters' external appearance from childhood to adulthood, both during their economic boom and bust periods. In this way, her message of behaving toward the girls as normal is supported by clothes as a mirror of the soul theme. It is possible to understand Ilanit's way on the background of the cultural context in which we live. At the macro level, we live in a modern era that idolizes the ideal of beauty as an entry ticket into society. At the micro level, the interviewee was a beautiful lady for whom aesthetics and beauty are high on her list of priorities. The birth of two daughters with disabilities seems to have burst her illusions with regard to daughters for which she had hoped. Meticulous attention to external appearance was instilled into the girls and because part of their conduct.

Her close family circle's support played an important role in structuring her parenting experience. The grandmother's unconditional and non-judgmental devotion strengthened the mother's internal resources [24]. Thanks to her involvement, the mother managed to maintain a balance between her intense load bringing up her daughters and building a personal life including going out, entertainment, trips abroad, developing a relationship and even maintaining a social circle. The mother's siblings filled the gap that was created when their mother passed away.

It appears that her perception of her role as a mother derived from the norms upon which she was brought up. It is reasonable to assume that the example of a devoted mother that she saw in her childhood, and afterwards, influenced her life role in motherhood. Despite the fact that the totality of her maternal role demanded a high price from her in terms of profession and self-fulfillment, she never gave up on her daughters and never placed them in frameworks outside the home. Her desire to keep them under her wing is also expressed in the semantics of her words. She repeatedly called them “*my girls*”. As such, she stopped the time clock at their childhood. Subconsciously, she dictated time according to her individual internal clock. It appears that this process derives from an unwillingness to deal with her daughters' future, which is one of the known strategies employed by parents of children with special needs in order to cope [37].

Turning to religion without any need to become newly religious, was fruitful ground for all the mother's messages and insights that emerged from the themes. She and her daughters were welcomed, without any judgment regarding the girls' disabilities or her role as a mother, into the Rabbi's circle. She did not even have to change her dress code in order to be treated fairly. It appears that the faith in “*the ways of God being hidden*” saved her from having to deal with the question “*why did this happen to me?*” She thanked God at every opportunity and as such reinforced the message that “*everything will be okay*”.

So far, the internal circles that helped her parenting experiences have been described. However, wider support circles were unsatisfactory for her. She only praised an isolated number of frameworks, those that provided focused answers to her daughters' difficulties. However, she downplayed the other educational frameworks. Nor did she receive appropriate support from the welfare system. The frequent changes to social workers and their inability to respond every time she turned to them, made her understand that if she did not do things for her daughters, no one else would. She encountered limited budgets and bureaucratic obstacles and therefore, at a certain point she no longer viewed the welfare system and a source of support. Nevertheless, she continued her link with them so that her daughters would receive benefits to which they are entitled. Even their place of employments is not perceived as appropriate for her daughters, particularly the younger one. She identified the educational staff's tiredness. However she did not give up on this framework for her daughters because she understood that belonging to it gave her a modicum of relief from the burden.

In conclusion, the parenting experience of a single mother to two daughters with cognitive disabilities, as expressed in this research, is complex and fraught with challenges at life's junctions. It is an infinite process of changes demanding flexibility and internal work, adjustments and never ending dealing with many insoluble conflicts: Conflicts that move between guilt and acceptance, total devotion and loss of self-fulfillment, accepting one's children's differences and the desire to cover up their disabilities, worry (for the future) and hope and optimism (glass half full), separate experiences and infinite dependency etc. Maintaining a balance between these conflicts was possible thanks to the mother's internal resources and emotional strength. Family support allowed these same internal sources time to recharge and as such to stabilize this special parenthood.

This research focused on a mother's parenting experience because more often mothers are the ones who take upon themselves the role of bringing up their children. The task becomes heavier when a mother has to deal with parenting two children with special needs. Exposing the parenting process allows to divert the focus from problems that arise in this special type of parenthood, in the direction of positive powers and resources that exist in close proximity. When educationalists and other professionals (counselors, social workers, and therapists) who work in partnership with families of children with special needs in general and mothers of two children with special needs in particular, we must supply them with information, guidance and advice sensitively and without judgment. Adjusting intervention processes for mothers and family systems is likely to result in trusting relationship with professionals and strengthen mothers' feelings of self-efficacy and as such lead to cooperation between mothers and educational teams in the long term. These steps are likely to be an additional source of support in mothers' coping processes.

Research limitations: The current study is based on the life story of a mother to two daughters with cognitive disabilities. This research is too narrow and does not allow one to reach conclusions about the general population. It is possible that other life stories would raise additional ways of coping. In addition, had this interview taken place twenty years earlier, when the children were in their teens, it is possible that different themes would have emerged.

Proposals for additional studies: Professional literature contains very few studies that deal with parenting experiences of mothers of two children with special needs and even less with single mothers. A wider study examines and compares demographic, cultural, social and personality data of mothers to children with disabilities, is likely to illustrate parenting styles and ways of coping.

## Footnotes

1 The national association of restoration people with mental retardation in Israel.
